# Accuracy of AI-Assisted Methods in Preoperative Templating Compared to the Standard X-Ray-Based Surgeon-Performed Templating in Total Hip Replacement Surgery: A Systematic Review and Meta-Analysis

**DOI:** 10.7759/cureus.95328

**Published:** 2025-10-24

**Authors:** Osamah Al-Zubaidi, Aiman Alzuabidi, Ban Alkurdi, Dina Alkurdi, Nayef Aslam-Pervez

**Affiliations:** 1 Plastic Surgery, Hull University Teaching Hospitals NHS Trust, Hull, GBR; 2 Emergency Department, Royal United Hospital, Bath, GBR; 3 Dentistry, Independent Research, Hull, GBR; 4 Trauma and Orthopaedics, Hull University Teaching Hospitals NHS Trust, Hull, GBR

**Keywords:** ai, artificial intelligence, pre-operative planning, preoperative templating, total hip arthroplasty: tha, total hip replacement (thr)

## Abstract

Accurate preoperative templating in total hip arthroplasty (THA) improves the procedure’s precision, shortens its duration, and reduces complications such as instability and dislocations, prosthesis loosening, and loss of bone stock, among others. The current standard method uses X-ray images to manually determine implant size; however, the emergence of artificial intelligence (AI) is gaining increasing interest in its use for preoperative templating.

This systematic review and meta-analysis aims to assess the difference in accuracy between the standard and AI methods for preoperative templating in regards to implant size. A systematic search of electronic databases identified 36 papers related to AI in preoperative planning in THA. Of these, 9 studies met the inclusion criteria and cumulatively yielded 1049 patient for comparison. Statistical analysis was performed using OpenMetaAnalysis software, utilising a random effects model with reported results at 95% confidence intervals. Results showed the odds for exact size match using an AI-assisted method were 4.163 times higher than the standard manual method in predicting an acetabular cup component (OR=4.163, P<0.001) and 3.672 times higher in predicting a femoral stem component (OR=3.672, P<0.001). Moreover, operative time following the use of the AI-assisted method was 9.2 minutes less than operations following the surgeon-performed method (MD=-9.2, P=0.027), although this was reduced to 4.35 minutes when the source of heterogeneity was removed (MD=-4.35, P=0.025). A key limitation of this study is that all the papers identified in the literature and included in this meta-analysis originated from China, thus limiting generalisability to other healthcare systems and populations. It was concluded that AI-assisted preoperative templating is significantly more accurate than the standard templating method currently used in clinical practice in predicting implant size in total hip arthroplasty and it helps to reduce operating time, although a high level of evidence from different centres worldwide is still lacking to validate its use in clinical practice.

## Introduction and background

Hip preoperative templating is the process of estimating implant size and positioning in total hip arthroplasty (THA) using preparatory imaging [1]. Accurate templating enhances surgical precision, shortens operative time, and reduces complications such as instability and dislocation, prosthesis loosening and bone stock loss [2,3].

Traditionally, templating was performed manually by overlaying transparencies of magnified implants on radiographs to determine the optimal implant size [4]. The introduction of Picture Archiving and Communication Systems (PACS) has since enabled digital templating, which uses external calibration objects to calculate radiographic magnification [5]. In digital radiography, computer software adjusts templates for magnification [6], after which the surgeon identifies anatomical landmarks, evaluates image quality, defines mechanical references, and positions acetabular and femoral components to achieve a final plan [1]. While this 2D templating method remains the clinical standard [7], it is limited by the intrinsic complexity of the hip as a 3D joint [7]. Errors may arise from magnification, patient positioning, or biomechanical measurements such as femoral offset, which can be affected by hip flexion or rotation [7].

CT-based templating provides a more detailed three-dimensional assessment but involves a labour-intensive workflow, including image segmentation, pelvic correction, deformity recognition, and postoperative simulation. As a result, it is time-consuming and requires collaboration between engineers, programmers, and surgeons, limiting its routine use [8].

Artificial intelligence (AI) has recently emerged as a potential solution, offering automated data organisation, implant sizing, and surgical guidance [9]. AI-HIP software (Beijing Changmugu Medical Technology Co., Ltd., Beijing, China) is one such 3D CT-based system that applies deep learning algorithms to segment images and support preoperative planning. Although increasingly adopted, its role in orthopaedics has not yet been fully validated [10].

This systematic review and meta-analysis, therefore, aims to compare the accuracy of AI-assisted 3D preoperative templating with the conventional 2D approach.

## Review

Methods 

**Figure 1:**
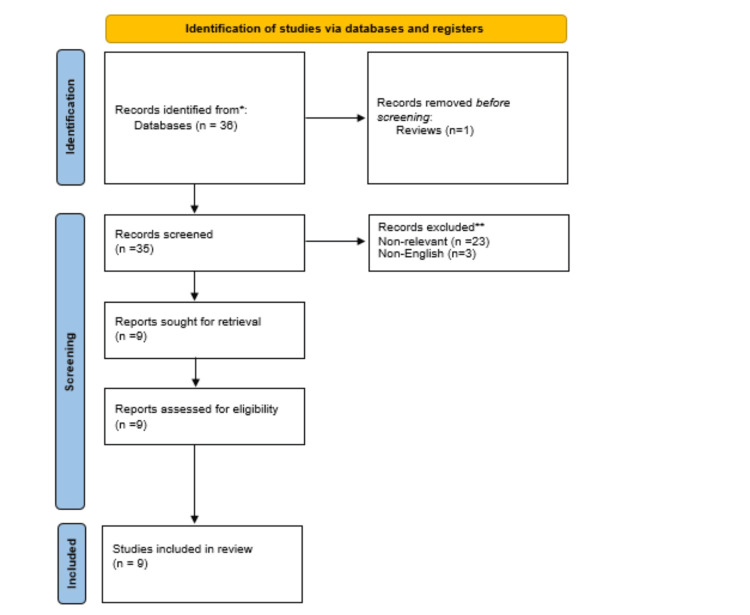
PRISMA flow chart followed in this meta-analysis PRISMA: Preferred Reporting Items for Systematic Reviews and Meta-Analyses Source: [11]

This systematic review and meta-analysis were performed as per the Preferred Reporting Items for Systematic Reviews and Meta-Analyses (PRISMA) statement standards (Figure 1) [11].

Eligibility Criteria

All studies, including randomised and non-randomised trials and observational studies, comparing the accuracy of preoperative templating using an AI-assisted method to the preoperative templating using a standard X-ray-based, human-conducted method in primary and revision total hip replacement operations. Only studies in English were included. There was no restriction on age, comorbidities, indication for total hip replacement, place of study, or size of study.

Exclusion Criteria

Duplicates, abstracts, non-comparative studies, and studies that were not in English and any study that failed to report on the authors’ defined primary outcome measure, which was the exact match in size of the preoperative template with the actual implant size used intraoperatively, were excluded.

Outcome Measures

The primary outcome was the exact match in size of the preoperative template with the actual implant size used intraoperatively. Operative time was the secondary outcome.

Literature Search Strategy

Two authors independently searched the published electronic literature in Google Scholar, PubMed, and MEDLINE. The last search was conducted on 27/5/2025 at 15:39. The search terms included “AI”, "artificial intelligence", ”THR”, "hip replacement", "hip arthroplasty", “THA”, “templating”, “template”. The terms were collated with adjuncts, including ‘and’ as well as ‘or’. The authors also searched the bibliographical lists of relevant articles to optimise article retrieval.

Selection of Studies

Two authors independently evaluated the titles and abstracts of all studies, and those meeting the eligibility criteria were subsequently assessed for their full texts. Any discrepancy in selection was resolved by consultation with a third author.

Data Extraction and Management

A data extraction spreadsheet based on Cochrane’s data collection form for interventional reviews was drafted on Microsoft Excel (Microsoft Corporation, Redmond, WA, US). The information collected included: study year, study design, total number of patients, number of patients who had an exact match using AI and standard templating for acetabular and femoral components, and operative time. The extraction form was pilot tested on randomly selected articles initially before undergoing adjustments. Two authors independently extracted and entered the data, with any ambiguity being resolved by discussion. The included papers and extracted data are detailed in Table 1.

**Table 1:** Amalgamation of included papers demonstrating main author, year, study design and results Sources: [8-10,12-17] n: sample size, AI: AI-assisted templating, Man: manual performed templating, NR: not reported

		Acetabulum Size Match /n		Stem Size Match /n		Operation Time (Minutes)	
Author, year	Study design	AI	Man	AI	Man	AI	Man
Huo et al, 2021 [12]	Randomised controlled trial	44/59	24/59	42/59	29/59	NR	NR
Ding et al, 2021 [10]	Crossover retrospective	297/316	206/316	277/316	186/316	NR	NR
Chen et al, 2022 [8]	Randomised controlled trial	40/60	20/60	33/60	19/60	106.83 ± 18.20	109.58 ± 21.98
Wu Zhau, 2023 [13]	None-randomised retrospective	51/95	25/66	61/95	29/66	78.5±18.5	83.6±18.7
Wu Yang et al, 2023 [14]	None-randomised retrospective	19/34	8/27	23/34	11/27	78.8±16.9	83.4±17.5
Xie et al, 2024 [9]	Crossover retrospective	138/164	105/164	NR	NR	NR	NR
Anwar et al, 2024 [15]	Crossover retrospective	78/117	36/117	77/117	44/117	NR	NR
Yang et al, 2024 [16]	Randomised retrospective	169/220	89/220	175/220	101/220	43.22 ± 12.65	68.36 ± 14.45
Zheng et al, 2025 [17]	Randomised controlled trial	22/29	12/27	24/29	16/27	95±13.74	100±26.51

Data Synthesis

OpenMetaAnalysis software (https://openmetaanalysis.github.io/) was utilised to conduct data synthesis with a random effects model being adapted and forest plots reported at 95% confidence intervals (CI). For dichotomous outcome data, the odds ratio was used. This enabled the assessment of the AI-assisted templating method against the standard X-ray-based, surgeon-conducted templating method. For continuous data, the mean difference was utilised to assess the difference in operative time between the two groups.

Methodological Quality and Risk of Bias

The Newcastle-Ottawa Scale (NOS) was used to assess the quality of all non-randomised retrospective studies (Table 2) [18]. It uses a star system with three different domains: selection of study groups, comparability of groups, and the exposure/outcome of interest. It allows for the allocation of a maximum of nine stars for each study. The Cochrane risk of bias tool (The Cochrane Collaboration, London, England, UK) was reserved for the assessment of randomised and crossover trials (Table 3).

**Table 2:** Risk-of-bias assessment as per the Newcastle-Ottawa Scale Source: [13,14]

Author, year	Selection	Comparability	Exposure
Wu, Zhau, et al 2023 [13]	****	**	***
Wu, Yang, et al, 2023 [14]	****	**	***

**Table 3:** Risk-of-bias assessment as per the revised Cochrane risk-of-bias tool Risk-of-bias assessment as per the revised Cochrane risk-of-bias tool, where 1 = low risk, 2 = some concern, and 3 = high risk [8-10,12,15-17]. A detailed assessment can be found in the Appendices. D: domain, N/A: not applicable

Author, year	D1: Risk of bias arising from the randomisation process	S: Risk of bias arising from period and carryover effects (crossover studies)	D2: Risk of bias due to deviations from the intended interventions (effect of assignment to intervention)	D3: Risk of bias due to missing outcome data	D4: Risk of bias in measurement of the outcome	D5: Risk of bias in the selection of the reported result	Overall
Xie et al, 2024 [9]	1	1	1	1	1	1	1
Zheng et al, 2025 [17]	1	N/A	1	1	1	1	1
Anwar et al, 2024 [15]	1	1	1	1	1	1	1
Yang et al, 2024 [24]	1	N/A	1	1	1	1	1
Huo et al, 2021 [12]	1	N/A	1	1	1	1	1
Ding et al, 2021 [10]	1	1	1	1	1	1	1
Chen et al, 2022 [8]	1	N/A	1	1	1	1	1

Assessment of Heterogeneity

Heterogeneity for all studies was assessed by a dual method of using the Cochran Q test (v2) as well as calculating the I² score. This was interpreted along the following scale: 0-25% low heterogeneity; 25%-75% moderate heterogeneity; and 75%-100% high heterogeneity.

Results

The online literature search retrieved 36 articles. These were screened independently by two authors who excluded duplicates, abstracts, non-comparative studies, studies that were not in English, and any study that failed to report on the author-defined primary outcome measure, which was the exact match in size of the preoperative template with the actual implant size used intraoperatively. A total of nine studies met the inclusion criteria for quantitative synthesis and cumulatively yielded 1049 patients for comparison [8-10,12-17].

AI-Assisted Templating Accuracy

**Figure 2:**
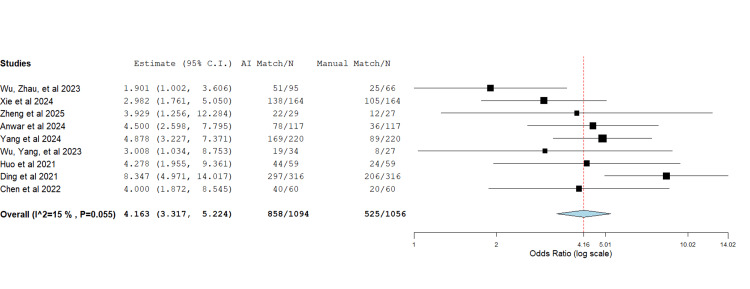
Forest plot showing the odds ratio of the acetabular size match between the preoperative planned size and the actual size used intraoperatively The use of AI in preoperative planning was superior to the standard manual method. OR= 4.163, P<0.001, heterogeneity: I2 = 15%, P= 0.055 Source: [8-10,12-17]

Acetabular component: Nine studies overall reported on the exact match in size of the preoperative template with the actual implant size used intraoperatively for the acetabular cup (Figure 2) [8-10,12-17]. The data showed a heterogeneity of I2 (15%) and a P-value of 0.055. The overall accuracy of using the AI-assisted method was 4.163 times higher than the standard manual method in predicting the acetabular cup component (OR=4.163, P<0.001).

**Figure 3:**
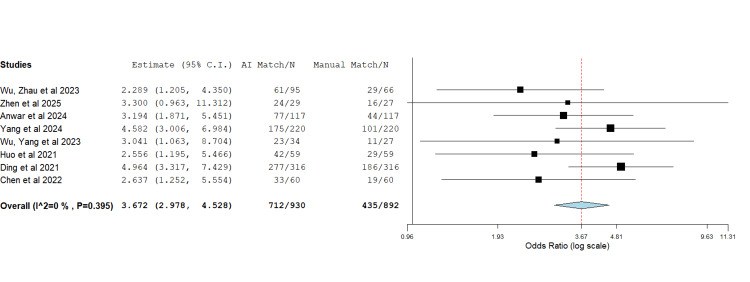
Forest plot showing the odds ratio of the femoral stem size match between the preoperative planned size and the actual size used intraoperatively The use of AI in preoperative planning was superior to the standard manual method. OR=3.672, P<0.001, heterogeneity: I2 = 0%, and P=0.395 Source: [8,10,12–17]

Femoral component: Eight studies reported on the exact match in size of the preoperative template with the actual implant size used intraoperatively for the femoral stem (Figure 3) [8,10,12-17]. The data showed a heterogeneity of I2 (0%) and a P-value of 0.395. The overall accuracy of using the AI-assisted method was 3.672 times higher than the standard manual method in predicting the femoral stem component (OR=3.672, P<0.001).

Operative Time

**Figure 4:**
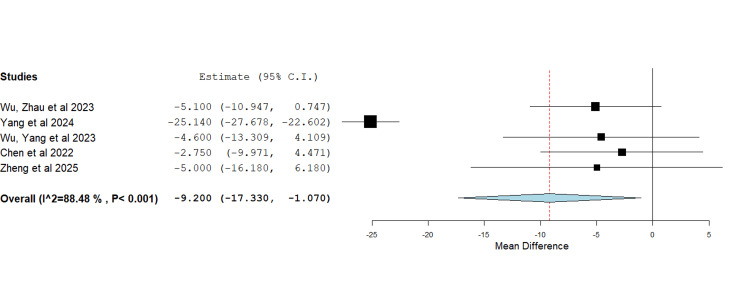
Forest plot showing the mean difference in operative time between operations in which AI preoperative planning was used and operations in which the standard manual method was used The use of AI in preoperative planning resulted in a shorter operative time compared to the standard manual method. MD=-9.2, P=0.027 heterogeneity: I2 =88.48%, P value < 0.001 Note that Yang et al. (2024) is an outlier and is the source of heterogeneity. Source: [8,13,14,16,17]

**Figure 5:**
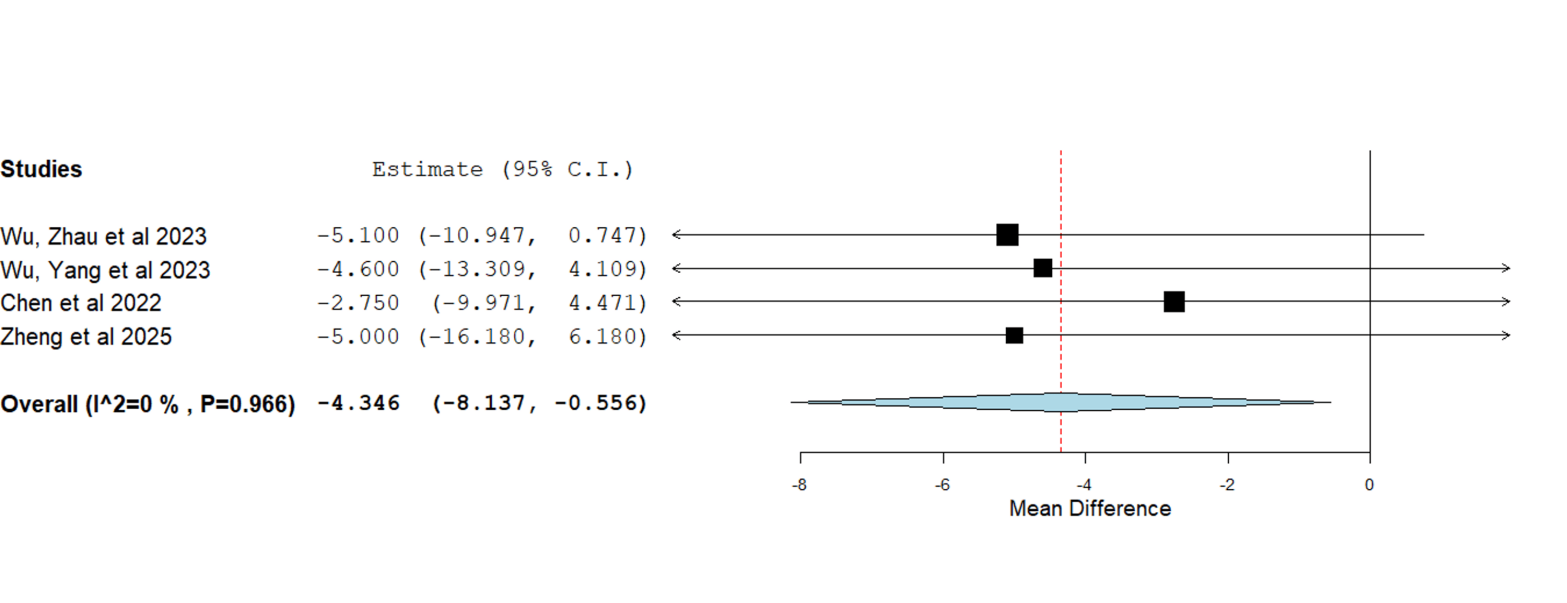
Forest plot showing the mean difference in operative time between operations in which AI preoperative planning was used and operations in which the standard manual method was used after removal of heterogeneity The use of AI in preoperative planning resulted in a shorter operative time, as compared to the standard manual method. MD=-4.35, P=0.025 heterogeneity: I2=0%, P-value < 0.966 Source: [8,13,14,17]

Five studies reported on the operative time using the AI-assisted and surgeon-performed methods (Figure 4) [8,13,14,16,17]. The initial meta-analysis of data showed a significant heterogeneity of 88.48% and a P-value of < 0.001. Operation time following the use of the AI-assisted method was 9.2 minutes less than operations following the surgeon-performed method (MD=-9.2, P=0.027). However, as evident from the forest plot in Figure 4, the Yang et al. paper was identified as an outlier and the source of heterogeneity [16]. After excluding the source of heterogeneity, i.e., Yang et al. [16], the analysis showed that the operation time following the use of the AI-assisted method was 4.35 minutes less than operations following the surgeon-performed method (MD=-4.35, P=0.025) with heterogeneity of 0% and P-value 0.966 (Figure 5).

Limitations

In this meta-analysis, only three studies were prospective randomised controlled trials. Thus, higher-level evidence remains necessary to validate the role of AI in preoperative templating. Additionally, all included studies were conducted in China, limiting the generalisability to other healthcare systems and populations.

Discussion

AI is a rapidly developing technology, and its use in medical practice has been gaining increasing interest in recent years. However, its role and validity remain to be fully established. Our meta-analysis aimed to assess the superiority of AI-assisted preoperative templating over the standard X-ray-based surgeon-conducted method and to identify existing gaps in the literature. This review builds upon the preceding systematic review performed by Mozafari et al. [19] by including three additional studies and performing a meta-analysis.

In total, nine studies [8-10,12-17] met the inclusion criteria. The pooled odds ratio assessment demonstrated a significant increase in the accuracy of preoperative templating in total hip replacement (THR) when AI-assisted methods were employed. Moreover, the mean difference analysis showed reduced operative time with AI use. The superiority of AI accuracy is likely attributable to the advantage of 3D CT imaging over conventional 2D radiographs [7]. Notably, Huo et al. reported no significant difference in accuracy between AI-assisted and surgeon-performed templating when both utilised 3D models, though AI templating remained significantly quicker by 28.1 minutes [12]. Similarly, a shorter operative time may result from enhanced accuracy, which reduces intraoperative adjustments [2]. Nevertheless, our operative time analysis showed high heterogeneity, likely reflecting differences in surgical approach, as Yang et al. [16] was the only paper in which the direct anterior approach to the hip was used, whilst the posterolateral approach was used in all other papers [8,13,14,17]. To control this, another meta-analysis excluding the source of heterogeneity was performed, and both results were reported.

Although risk-of-bias assessment revealed overall good quality using the NOS and the Cochrane risk-of-bias tool, only three studies were prospective randomised controlled trials. Thus, higher-level evidence remains necessary to validate the role of AI in preoperative templating. Additionally, all included studies were conducted in China, limiting the generalisability to other healthcare systems and populations.

Beyond accuracy and operative efficiency, the clinical implications of AI-assisted templating merit attention. Greater preoperative precision could improve implant positioning, lower the risk of complications, and potentially reduce revision rates [2,3], thereby enhancing long-term patient outcomes. Reduced operative time may also translate into lower intraoperative infection risk and improved operating theatre utilisation. However, the adoption of AI and 3D CT-based methods carries economic considerations. While operative time savings may reduce costs, the increased expense of advanced imaging and AI software may offset these benefits, warranting future cost-effectiveness analyses.

The integration of AI into surgical workflows also raises practical and ethical questions. Surgeons may require training to effectively interpret AI-generated templates, but AI could simultaneously reduce inter-operator variability, particularly among less experienced surgeons. Ethical and regulatory concerns, including data security, algorithm transparency, and validation across diverse patient groups, must be addressed before widespread implementation.

The authors, therefore, recommend the consideration of the use of AI-assisted preoperative templating for THA surgeries and the adoption of large, multicentre, prospective, double-blinded randomised control studies to fill the gap and gain a high level of evidence to support the introduction of the use of AI-assisted preoperative templating in total hip replacement surgeries in standard clinical practice. We also recommend further studies to assess the cost-effectiveness and the long-term outcomes such as patient satisfaction, implant survival, and rate of revision.

## Conclusions

AI-assisted preoperative templating showed a significant increase in accuracy and resulted in shorter operative duration when compared to the standard X-ray-based, surgeon-performed preoperative templating in total hip replacement, although a high level of evidence is still lacking. The adoption of large, multicentre, prospective, double-blinded, randomised control studies is recommended to fill the gap and gain a high level of evidence to support the introduction of the use of AI-assisted preoperative templating in total hip replacement surgeries in standard clinical practice.

## Figures and Tables

**Figure 6:**
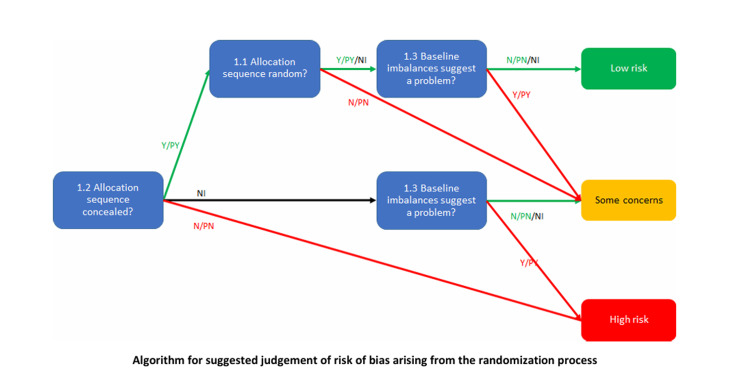
Flow chart for the suggested judgement for Domain 1 in the Cochrane risk of bias assessment tool

**Figure 7:**
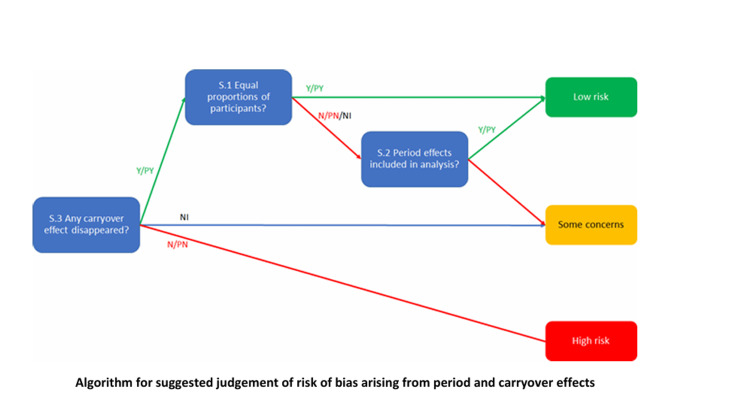
Flow chart for the suggested judgement for Domain S in the Cochrane risk of bias assessment tool

**Figure 8:**
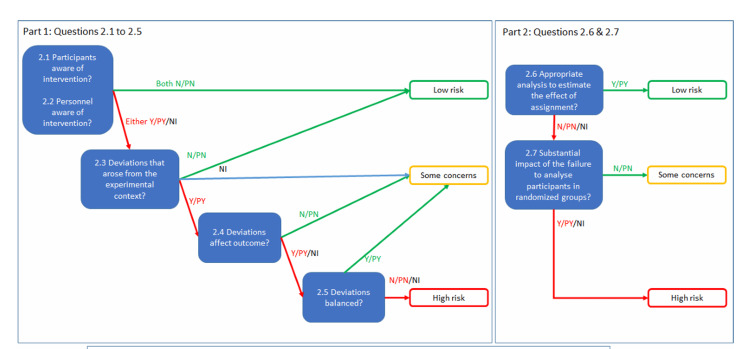
Flow chart for the suggested judgement for Domain 2 in the Cochrane risk of bias assessment tool

**Figure 9:**
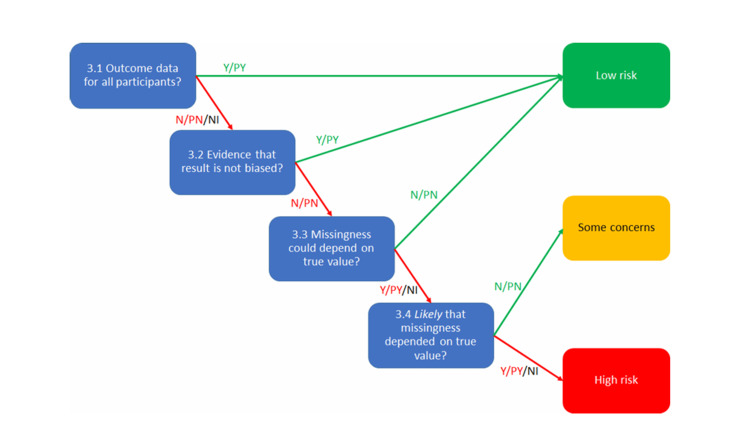
Flow chart for the suggested judgement for Domain 3 in the Cochrane risk of bias assessment tool

**Figure 10:**
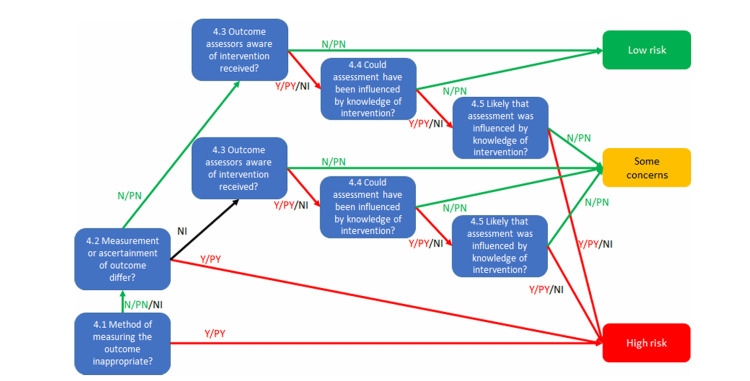
Flow chart for the suggested judgement for Domain 4 in the Cochrane risk of bias assessment tool

**Figure 11:**
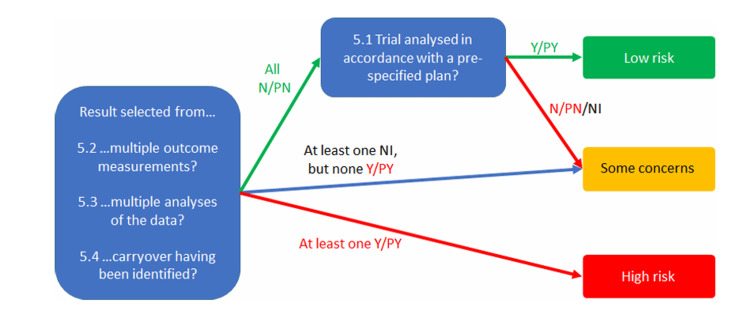
Flow chart for the suggested judgement for Domain 5 in the Cochrane risk of bias assessment tool

## Appendices

The process of risk of bias assessment using the Cochrane risk of bias assessment tool is detailed in Tables 4-9 and Figures 6-11.

**Table 4:** Domain 1: Risk of bias arising from the randomisation process Domain 1: Risk of bias arising from the randomisation process [8-10,12,15-17] Y=Yes, N=No

Author, year	1.1 Was the allocation sequence random?	1.2 Was the allocation sequence concealed until participants were enrolled and assigned to interventions?	1.3 Did baseline differences between intervention groups at the start of the first period suggest a problem with the randomisation process?
Xie et al, 2024	Y	Y	N
Zheng et al, 2025	Y	Y	N
Anwar et al, 2024	Y	Y	N
Yang et al, 2024	Y	Y	N
Huo et al, 2021	Y	Y	N
Ding et al, 2021	Y	Y	N
Chen et al, 2022	Y	Y	N

**Table 5:** Domain S: Risk of bias arising from period and carryover effects Domain S: Risk of bias arising from period and carryover effects [9,10,15] Y=Yes

Author, year	S.1 Was the number of participants allocated to each of the two sequences equal or nearly equal?	S.3 Was there sufficient time for any carryover effects to have disappeared before outcome assessment in the second period?
Xie et al, 2024	Y	Y
Anwar et al, 2024	Y	Y
Ding et al, 2021	Y	Y

**Table 6:** Domain 2: Risk of bias due to deviations from the intended interventions (effect of assignment to intervention) Domain 2: Risk of bias due to deviations from the intended interventions (effect of assignment to intervention) [8-10,12,15-17] Y=Yes, PY=Probably Yes, N=No, NI=No Information, N/A=Non-Applicable

Author, year	2.1. Were participants aware of their assigned intervention during each period of the trial?	2.2. Were carers and people delivering the interventions aware of participants' assigned intervention during each period of the trial?	2.3. If Y/PY/NI to 2.1 or 2.2: Were there deviations from the intended intervention that arose because of the trial context?	2.6 Was an appropriate analysis used to estimate the effect of assignment to intervention?
Xie et al, 2024	N	N	N/A	Y
Zheng et al, 2025	N	PY	N	Y
Anwar et al, 2024	N	N	N/A	Y
Yang et al, 2024	N	N	N/A	Y
Huo et al, 2021	N	N	N/A	Y
Ding et al, 2021	N	N	N/A	Y
Chen et al, 2022	N	NI	N	Y

**Table 7:** Domain 3: Risk of bias due to missing outcome data Source: [8-10,12,15-17] Y=Yes, PY=Probably Yes, N=No, NI=No Information, N/A=Non-Applicable

Author, year	3.1 Were data for this outcome available for all, or nearly all, participants randomised?	3.2 If N/PN/NI to 3.1: Is there evidence that the result was not biased by missing outcome data?	3.3 If N/PN to 3.2: Could missingness in the outcome depend on its true value?
Xie et al, 2024	Y	N/A	N/A
Zheng et al, 2025	N	NI	N
Anwar et al, 2024	Y	N/A	N/A
Yang et al, 2024	Y	N/A	N/A
Huo et al, 2021	Y	N/A	N/A
Ding et al, 2021	Y	N/A	N/A
Chen et al, 2022	Y	N/A	N/A

**Table 8:** Domain 4: Risk of bias in measuring the outcome Source: [8-10,12,15-17] Y=Yes, PY=Probably Yes, N=No, NI=No Information, N/A=Non-Applicable

Author, year	4.1 Was the method of measuring the outcome inappropriate?	4.2 Could measurement or ascertainment of the outcome have differed between interventions within each sequence / group?	4.3 If N/PN/NI to 4.1 and 4.2: Were outcome assessors aware of the intervention received by study participants?	4.4 If Y/PY/NI to 4.3: Could the assessment of the outcome have been influenced by knowledge of the intervention received?
Xie et al, 2024	N	N	NI	N
Zheng et al, 2025	N	N	NI	N
Anwar et al, 2024	N	N	NI	N
Yang et al, 2024	N	N	NI	N
Huo et al, 2021	N	N	NI	N
Ding et al, 2021	N	N	NI	N
Chen et al, 2022	N	N	NI	N

**Table 9:** Domain 5: Risk of bias in the selection of the reported result Source: [8-10,12,15-17] Y=Yes, PY=Probably Yes, N=No, NI=No Information, N/A=Non-Applicable

Author, year	5.1 Were the data that produced this result analysed in accordance with a pre-specified analysis plan that was finalised before unblinded outcome data were available for analysis??	5.2 Is the numerical result being assessed likely to have been selected, on the basis of the results, from multiple eligible outcome measurements (e.g. scales, definitions, time points) within the outcome domain?	5.3 Is the numerical result being assessed likely to have been selected, on the basis of the results, from multiple eligible analyses of the data?	5.4 Is a result based on data from both periods sought, but unavailable on the basis of carryover having been identified? (Crossover studies)
Xie et al, 2024	Y	N	N	N
Zheng et al, 2025	Y	N	N	N/A
Anwar et al, 2024	Y	N	N	N
Yang et al, 2024	Y	N	N	N/A
Huo et al, 2021	Y	N	N	N/A
Ding et al, 2021	Y	N	N	N
Chen et al, 2022	Y	N	N	N/A
